# Supplementary material for: Individual and intimate-partner factors associated with cervical cancer screening in Central Uganda
Source: PLoS One. 2022 Sep 15;17(9):e0274602. doi: 10.1371/journal.pone.0274602 (PMC9477300; doi:10.1371/journal.pone.0274602)
Supplement: Attachment — Submitted filename: PLOS HPV.docx [file pone.0274602.s004.docx]

**Comments**

This research article signifies the women and the partner’s factors associated with the cervical HPV screening in the underprivileged population of Central Uganda having high burden of the cancer. The article has already been through the major revision and the author though has claimed to have addressed the major observations marked previously. The study is reasonably constructed and can be published but the author has to address the major observations noted herein again as follow.

**Title**

Title of the article has not been revised as suggested by both the previous reviewers. Streamline the literature presented with suitable title.

The red highlights in the “Revised Manuscript with Track Changes“ in the pdf file mostly are the same as presented in the original submission.

Improve the expression of English suggested by the Reviewer 1 as the red highlights are not changes but are consistent with the original submission.

**Abstract**:

P2L3 Replace capital M in male with small letter. Revise English language for better understanding.

**Background**

Track changes appearing red are the same as the original submission.

Highlight the knowledge gap and rationalize your study as previously suggested

**Methods**

Cite Reierson Draugalis et al. in the references in the sub heading of “Sample size and sampling procedure.

The author adopted the questionnaire from the previous studies. Was the translated questionnaire checked for reliability and validity? And what about the internal consistency, pilot test and Cronback’s alpha?

**Results**

Tables are not presented as three-line tables.

**Discussion**

Adequate

**Conclusion**

Adequate

**References**

Format of references not rectified and missing journal name, volume, issue and page number should be mentioned (Ref 1, Ref 14, Ref 21, Ref 28, Ref 34, Ref 38, Ref 49, Ref 43, Ref 46, Ref 50, Ref 56, Ref 58 and others
