# Supplementary material for: Individual and intimate-partner factors associated with cervical cancer screening in Central Uganda
Source: PLoS One. 2022 Sep 15;17(9):e0274602. doi: 10.1371/journal.pone.0274602 (PMC9477300; doi:10.1371/journal.pone.0274602)
Supplement: Attachment — Submitted filename: Rebuttal-letter-Plos-20-8-2022.docx [file pone.0274602.s005.docx]

**Response letter**

| **Date** | 18 August 2022 |
| --- | --- |
| **To:** | PLOS ONE em@editorialmanager.com |
| **From:** | "Alone Isabirye" aloneisab@gmail.com |
| **Subject:** | Response to review comments of our manuscript submitted to PLOS ONE (PONE-D-20-2133110R1) |

Individual and intimate-partner factors of cervical cancer screening in Central Uganda
Alone Isabirye,

Dear Editor,

Thank you for your reply regarding my manuscript "Individual and -intimate-partner factors of cervical cancer screening in Central Uganda". I am grateful for your and the reviewers’ comments. I have revised and modified the manuscripts according to your and reviewer’s comments. As a consequence, I provide a revised manuscript with your and the reviewers’ suggestions integrated therein:

**Response to editor’s comments**

The previous Reviewer's comments (Reviewer 2) were not satisfactorily addressed. Another reviewer was invited and the new comments (Reviewer 3) are also not very encouraging. I urge you to carefully address all the concerns by responding to each query of previous as well as new reviewer. The publication at this stage depends upon the quality of response from you.

All the comments are clear and self-explanatory, but if you need further clarity, please feel free to contact us.

Thank you for coordinating the review process of my manuscript. A lot of care has been exercised to ensure that satisfactory revision and response to the comments is attained. The responses to the comments and revisions are provided below and in the manuscript respectively.

**Response to reviewer 3’s comments**

This research article signifies the women and the partner’s factors associated with the cervical HPV screening in the underprivileged population of Central Uganda having high burden of the cancer. The article has already been through the major revision and the author though has claimed to have addressed the major observations marked previously. The study is reasonably constructed and can be published but the author has to address the major observations noted herein again as follow.

**Title**

Title of the article has not been revised as suggested by both the previous reviewers. Streamline the literature presented with suitable title.

The title was revised to suit the literature presented in the article.

The red highlights in the “Revised Manuscript with Track Changes“ in the pdf file mostly are the same as presented in the original submission.

Thank you for the observation. Sections that were highlighted, and yet they were not affected by the changes were revisited and presented as normal text.

Improve the expression of English suggested by the Reviewer 1 as the red highlights are not changes but are consistent with the original submission.

Thank you for the observation. Sections that were highlighted and yet they were not affected by the changes were revisited and presented as normal text.

The manuscript was revised and language issues were fixed.

**Abstract**:

P2L3 Replace capital M in male with small letter. Revise English language for better understanding.

Upper-case “M” was replaced with lower-case “m”.

The manuscript was revised and language issues were fixed.

**Background**

Track changes appearing red are the same as the original submission.

Thank you for the observation. Sections that were highlighted and yet they were not affected by the changes were revisited and presented as normal text.

Highlight the knowledge gap and rationalize your study as previously suggested

The knowledge gap and rationale for the study were mentioned in the last paragraph of the background.

**Methods**

Cite Reierson Draugalis et al. in the references in the sub heading of “Sample size and sampling procedure.

Reierson Draugalis et al., was cited following the recommended format.

The author adopted the questionnaire from the previous studies. Was the translated questionnaire checked for reliability and validity? And what about the internal consistency, pilot test and Cronbach’s alpha?

Issues related to questionnaire validation and ensuring internal consistency (reliability) were explained. This involved pilot-testing the tool, and establishing its internal consistency by use of efficacious approaches like the Cronbach Alpha; these have also been sufficiently substantiated in the revised manuscript.

**Results**

Tables are not presented as three-line tables.

Results were presented as three-line-tables

**Discussion**

Adequate

The comment about the discussion section was appreciated.

**Conclusion**

Adequate

The positive comment about the conclusion was appreciated.

**References**

Format of references not rectified and missing journal name, volume, issue and page number should be mentioned (Ref 1, Ref 14, Ref 21, Ref 28, Ref 34, Ref 38, Ref 49, Ref 43, Ref 46, Ref 50, Ref 56, Ref 58 and others

All references were revisited, and sufficient detail was provided.

I hope that my modifications render my manuscript in its current form suitable for publication in PLOSONE

Yours sincerely,

Isabirye Alone

aloneisab@gmail.com
